# “Is it menopause or bipolar?”: a qualitative study of the experience of menopause for women with bipolar disorder

**DOI:** 10.1186/s12905-017-0467-y

**Published:** 2017-11-16

**Authors:** Tania Perich, Jane Ussher, Chloe Parton

**Affiliations:** 1 Clinical and Health Psychology Research Initiative (CaHPRI), School of Social Sciences and Psychology, Western Sydney University, Sydney, Australia; 2 School of Psychiatry, University of New South Wales, Sydney, Australia; 3 Translational Health Research Institute, Western Sydney University, Sydney, Australia

**Keywords:** Bipolar disorder, Menopause, women’s health, women’s mental health

## Abstract

**Background:**

Menopause can be a time of change for women and may be marked by disturbances in mood. For women living with a mental illness, such as bipolar disorder, little is known about how they experience mood changes during menopause. This study aimed to explore how women with bipolar disorder constructed mood changes during menopause and how this impacted on treatment decisions.

**Methods:**

Semi-structured interviews were undertaken with fifteen women who reported they had been diagnosed with bipolar disorder. Data was analysed using thematic analysis guided by a social constructionist framework.

**Results:**

Themes identified included ‘Constructions of mood change: menopause or bipolar disorder?’,‘ Life events, bipolar disorder and menopause coming together’; ‘Treatment choices for mood change during menopause’.

**Conclusions:**

The accounts suggested that women related to the experience of mood changes during menopause through the lens of their existing framework of bipolar disorder, with implications for understanding of self and treatment choices.

**Electronic supplementary material:**

The online version of this article (10.1186/s12905-017-0467-y) contains supplementary material, which is available to authorized users.

## Background

Bipolar disorder is a condition that affects around 1 % of the population [1]. Bipolar I disorder is characterised by at least one manic or mixed episode, whilst Bipolar II disorder is characterised by at least one hypomania episode and a history of at least one episode of major depression [2]. Depressive symptoms can be experienced three times more often than hypo/mania symptoms and even with treatment, people may experience mood symptoms up to 50% of the time [3]. In addition to depression and hypo/mania symptoms, around 75% of people with bipolar disorder will also meet criteria for another psychiatric condition [1]. Anxiety disorders are the most commonly reported, with panic disorder being the most prevalent of these [1]. The primary treatment for bipolar disorder is medication in the form of mood stabilizers, whilst psychotherapy such as CBT may also be used as an adjunct [4].

Although men and women are diagnosed with bipolar disorder at similar rates, some features of the illness, such as illness severity and illness course, may be worse for women than for men [5]. Panic disorder may be more common for women [6], whilst mixed episodes, depressive episodes [7] and rapid cycling may also occur more frequently [7, 8].

The experience of living with bipolar disorder may vary depending on the individual. Some may experience difficulties in a range of areas such as at work, education, relationships and in social lives, whilst others report that bipolar disorder opens up and creates opportunities for them, such as through expanded career paths and social networks [9]. A meta-data analysis of qualitative studies that explored the experience of symptoms and the diagnosis of bipolar disorder noted: ‘loss of control’, ‘disruption, uncertainty and instability’, ‘negative impact of symptoms across life and the experience of loss’, ‘negative view of self’, ‘struggling with the meaning of diagnosis’, ‘stigma’, and ‘acceptance and hope’ as some of the themes that arose within the lifelong process of living with bipolar disorder [10].

### Menopause and bipolar disorder

Reproductive-cycle events - such as the menstrual cycle [11], pregnancy, the postpartum period [12] and menopause [13] - may be marked by increased mood disturbance for women with bipolar disorder, with around 77% of women reporting worsening of mood at any of these times [14]. Other studies have also noted that 20% of women with bipolar disorder are at increased risk of mood episodes during menopause [15] and that women with bipolar disorder scored higher on depression and mania rating scales during the late transition or early menopause phase than women during early menopause [16]. It has also been report that midlife women with major depression and symptomatic menopausal transition had an increased risk of subsequent bipolar disorder compared to those with major depression alone [13].

Menopause can be a time of mood instability for women in the general population [17, 18], with some women experiencing mood changes such as irritability, depression and anxiety, that are attributed to menopause [19]. Medical treatment for severe menopausal symptoms may involve hormone treatment, however some women may also be prescribed non-hormonal treatments, such as anti-depressants [20]. Psychoeducational programmes, health education and promotion and cognitive behaviour therapy (CBT) have also been found to be an effective intervention for menopausal symptoms [21, 22].

However, negative mood change is not universal, and recent reviewers have cautioned against “over-pathologising” the menopause transition, as there is no clear evidence that depressive disorders occur more commonly in association with the menopause [23]. Menopause has been defined as a bio-psycho-socio-cultural transition [24, 25], with women’s experience of this life stage influenced by embodied change, social and cultural context, and the way they position psychological and embodied change during menopause [26–28]. Psychological distress during the menopausal transition has been associated with history of depression and sexual abuse, body mass index, and stressful life events at midlife [29, 30]. Increased prevalence of physical menopausal symptoms, such as hot flushes is also a risk factor [29, 31], with estradoil variability potentially enhancing emotional reactivity to psychosocial stress [32]. However, negative mood change is often attributed to menopause because of broader cultural discourse [33]. Women who have a negative view of menopause, and expect negative symptomatology, have been reported to be more likely to experience negative psychological symptoms [34, 35]. Equally, in cultural contexts wherein menopausal change is medicalised, women are more likely to report negative physical and psychological symptoms [36].

Little is known about how women with bipolar disorder construct mood changes during the time of menopause. These constructions may have implications for women’s experience of mood change during the menopausal transition, as well as for treatment and help seeking, and the nature or type of help that is sought. This study aimed to explore mood change as experienced during menopause for women with bipolar disorder using a qualitative design. The research questions were: How do menopausal women living with bipolar disorder construct their experience of mood change? What are the implications of these constructions for treatment seeking and their understanding of mood change?

## Methods

### Design

Qualitative data was analysed from semi-structured interviews with 15 women who had a diagnosis of bipolar disorder I or II and who had previously or were currently experiencing peri-menopause or menopause. A thematic analysis was employed to identify how the women construct experiences of mood change during menopause and the impact of these constructions on treatment.

### Participants and recruitment

Participants were recruited via advertising on social media, restricted to midlife women living in Australia, over a two-month period (March and April 2016). Inclusion criteria included women who had already experienced menopause as determined using Stages of Reproductive Aging Workshop (STRAW) criteria [37] and a previous diagnosis of bipolar disorder. Exclusion criteria included not having diagnosis of bipolar disorder, or having not yet entered menopause. Forty-six women completed an initial demographic information questionnaire which included questions about previous psychiatric diagnosis, illness course and menstrual status. Thirty-five women provided contact information for the qualitative interview component. Of these, six women were not eligible for the study, because of having reported a diagnosis other than bipolar disorder. Of those who were invited to participate, 15 women completed the qualitative interview component. Participant recruitment ceased once saturation of had occurred– no new themes in three successive interviews [38].

Mean age of the women who completed the interview was 52 years (range 46 to 60 years). The mean age of diagnosis of bipolar disorder was 39 years (sd 11.21). Nine participants were diagnosed after the age of 40 (60%), with 3 (20%) being diagnosed after the age of 50. Five participants were diagnosed with bipolar disorder (33%) during menopause.

Eight (53%) women were in the peri-menopause phase, defined using the STRAW criteria, whilst seven (47%) women were post-menopause. Five (33%) women reported they were diagnosed with bipolar I disorder and 10 (67%) with bipolar II disorder. Eleven (73%) were currently in a relationship (married or de-facto), and 14 (93%) identified as heterosexual. Seven (47%) women had completed a university degree (either undergraduate, post-graduate or PhD), whilst eight (53%) women were currently employed. Fourteen (93%) of the women were currently taking medication for bipolar disorder. All participants provided informed consent and the study was approved by the University Human Ethics Committee (HREC approval no. H11458). No incentives were offered to participants for taking part in the study.

### Procedure

The participants were initially asked to complete an online demographic survey. Questions included menstrual status, medication status, bipolar disorder type, age of onset and other co-occurring psychiatric diagnoses. Items related to menopause were phrased in order to determine menopause status in line with the Stages of Reproductive Aging Workshop (STRAW) criteria [37]. At the conclusion of the online survey, participants were invited to take part in a one-to-one interview about their experiences of menopause. Those who were eligible were then contacted via email or phone depending on their stated preference to organise an interview conducted over the phone.

Semi-structured interviews were conducted by the first author using the following questions: Can you tell me about your experience of menopause?; what features of menopause do/did you experience?; can you describe how these have/had an impact on you and your life; has menopause changed your bipolar disorder in any way? Follow up questions and prompts were framed using methods described by Magnusson and Marecek [39] which included questions being phrased as open-ended invitations to offer additional information. These included questions such as Can you give me a specific example of ..? [39]. Focused follow-up questions were also employed in order to elicit details about how the information may have related to menopause status [39]. All interviews were recorded for analytic purposes.

### Analysis

Participant interviews were transcribed using a professional transcription service and quality checked by the first author. The transcripts were analysed using inductive thematic analysis [40]. First, the authors became familiar with the data. This involved reviewing the transcribed data to check for accuracy, then repeatedly reading the data to identify potential codes. The second stage involved generating initial codes which involved working the data into meaningful groupings relevant to the research questions. The third stage involved using a coding framework where each line of the data was coded. The coded data was then used to form a coding summary from which themes were identified where the data was sorted into broader levels of themes and collated. In the fourth stage, themes were then defined and further refined by going back to review the data extracts for each theme. For the fifth stage, the themes were then reviewed and the themes were defined and named. A summary report which detailed the themes was then created and reviewed. Agreement was then met regarding the key themes that had been identified in the analysis. Data was viewed from a social constructionist perspective [41]. This approach posits a critical stance towards taken-for-granted knowledge, which is conceptualised as situated in and influenced by specific historical and cultural contexts and social processes [41]. More specifically, we drew on positioning theory [42], a social constructionist framework that posits that identity is constructed and negotiated in relation to the subject positions taken up by an individual, or the positions within which they are put by others [43]. We used this theory to look at how women made sense of menopausal symptoms, and how they positioned themselves as menopausal women, in relation to broader cultural discourses about menopause and midlife.

Three themes were identified in the analysis: ‘Constructions of mood change: menopause or bipolar disorder?’, ‘Life events, bipolar disorder and menopause coming together’; ‘Treatment choices for mood change during menopause’. Transcripts were de-identified and pseudonyms are used throughout the results presented below (Additional file 1).

## Results

### Constructions of mood change: Menopause or bipolar disorder?

The majority of women described experiencing mood changes around the time of menopause within the context of their bipolar disorder. Typically, women noted “bipolar symptoms” as occurring more often than before menopause “I’d probably have to say it’s been more frequent”, more severe “my lows are much lower”, and occurring in shorter and more intense bursts “more intense but not as long-lasting”. Several women spoke of a sensation of “...more rapid cycling through menopause”, with others noting mixed mood and more hypo/manic thoughts: "the feeling of my thoughts starting to race". The nature of mood change was always described as being negative, with many women reporting "not being able to cope, self-loathing", “regular thoughts of suicide” and “negative self-talk”. These accounts suggest that women constructed the experience of mood changes during this time of life through the lens of their existing framework of bipolar experience, rather than the lens of menopause, positioning themselves as ‘bipolar’ rather than ‘menopausal’.

Some women reported experiencing new types of bipolar symptoms during menopause, which were described as frightening: “that's a really, really scary thing”. For example, one participant said,

The type of depression that I suffer from is a very sad depression. I’m one of the uncontrolled weepy people….. but this is, there’s a urgency that comes with this [depression] that it’s almost like it doesn’t even come from me it’s like somebody behind me pushing me along. You know when you’re kids and then you’re holding hands, you hold hands and you’re running and you run downstairs or something like that and sometimes it’s scary, sometimes because it’s too fast you don’t know whether your feet can keep up and you could fall. That’s how it feels like, somebody else is either pushing or pulling me along and that’s too fast for me (Alyson).

The appearance of more severe mood symptoms during menopause was described as creating a destabilising effect and contributing to a sense of a loss of control, as described by Patty: “The anxiety is harder because the more anxious you get, the more anxious you get. And that it feeds itself. And you don’t really see an out for that”. For some women, the impact of the symptoms was so severe that it was experienced as incapacitating: "I can have an hour where I just have to lay on the lounge which, to be honest, is what I’m doing now".

Some women reported more feelings of anger and irritability that occurred without a specific trigger, which was different from how they had previously constructed their bipolar disorder and thus was seen as being different. For example, two women commented: "I just felt really angry at everybody" and “I just have this rage building up in me all the time”. For many women, these seemed to occur "in just in a split second" and with little warning “like Jekyll and Hyde”. The women positioned these mood changes, and by implication themselves, as uncharacteristic and reported subsequent difficulties within family relationships, with co-workers, or in the local community. As Marsha said:

I could go from placid, a placid person, to just this overwhelming anger in either, in just in a split second and it seemed to be the strangest things that were doing it. It just like little things that you’d normally brush off and I couldn’t brush them off and I kept saying to my husband ‘I have to go home, I can’t be here, I can’t be around people, I’ve got to go home’. I found that really difficult.

For many women, mood changes were described as having an impact on work and functioning, making day-to-day tasks, such as shopping, difficult to accomplish: “I drove there and I was in such a state I burst into tears, and it was total anxiety, shaking, and I had to turn around and come back without going to the shops.” The bipolar woman living through menopause was therefore positioned as unstable.

Menopause was positioned as the cause of the mood disturbance for some women \: “That only started [rapid cycling] when I started menopause; I never had that before.” However, other women’s accounts demonstrated a lack of clarity around whether change in mood was positioned as due to menopause or to bipolar disorder. In some of these accounts, the mood changes were attributed to bipolar disorder, as one participant said, “that’s probably associated with my bipolar.” However other women reported being unsure about how to make sense of their experiences. As Emily said in her interview: “I know since I've started menopause my symptoms seemed different, but whether that's a progression in the bipolar or whether that – the menopause affecting it, I'm not really sure”.

Carly also noted that her experience of bipolar depression was difficult to define, given that she was unclear as to her menopause status: “I’m not sure how far into menopausal I am or whether I’m just going through a rough trot with the depression – I don’t know”.

And Alyson spoke more generally of this life period, rather than mood symptoms specifically, “So I don't know, so this last eighteen months has had a monumental impact on my daily life but I can’t tell you with any degree of certainty whether any of the menopause symptoms have contributed to that”. In these accounts, women did not construct mood change as due to menopause or bipolar disorder, but rather adopted an ambivalent position in relation to the cause of their mood changes.

The women identified specific aspects of menopause that they associated with changes in mood. For example, physical symptoms of menopause were reported to have had an impact on symptoms of bipolar disorder for many interviewees, contributing to a sense of a loss of control over bipolar disorder during this time. In this example, the body was constructed as menopausal, whilst the mind, bipolar. As Annie described:

But when your body is doing its own thing and your brain is doing its own thing and you’re trying to get your brain into gear, and it’s like my brain is not under my control and my body is not under my control – well, who the hell am I and what am I doing and what am I – what’s going on? Everything doing its own sort of thing regardless of what you want.

Physical symptoms tended to be positioned as ‘menopause’ whilst psychological symptoms tended to be positioned as ‘bipolar disorder’ in the women’s accounts. Both the body (menopause) and the brain (bipolar disorder) were positioned as in control, serving to place the women in a passive position in relation to both menopause and bipolar disorder.

These accounts demonstrate that there was no clear framework within which women could make sense of mood change during menopause, and as a result, women oscillated between constructing symptoms as due to menopause or bipolar disorder. This may have implications for treatment seeking and choices regarding symptom management for women.

### A time of transition: Life events, bipolar disorder and menopause

Many women in the sample described significant life events and menopause coming together, in combination shaping their construction and experience of their usual bipolar disorder symptoms. For many, life events were described as having a greater impact than menopause or bipolar disorder on their experience of mood during this time of life. For example, Lane talked about multiple life events that coincided with menopause, saying,

Well, that’s a difficult one because I’ve gone through so much and I’ve come through where – see, I lost my mother about 18 months ago. And what had happened was I had actually taken six months – or I’ve resigned from my job, taken six months off to go down to Melbourne to be with my parents, with my husband’s blessing. And I only got there, Saturday night, Tuesday morning the first ambulance was called for Mum and she – we lost her a month later. And she was my father’s carer ‘cause he was a polio victim. And so, I stayed on to look after him and then of course we had to find a nursing home and sell the 58-year-old house and get rid of the contents and of course I was down in Melbourne and that’s when the marriage started to sort of fall apart, I think. So I think, perhaps, that was just like a snowball, really (Lane).

In this account, the multiple life events were positioned as leading to what Lane described as: “Just when I basically sat down and thought about everything that’s going on at once and just get to where I just can’t cope with it all. And I just go into a panic attack”. The magnitude and “snowball” nature of these significant stressors, including parental illness, geographical relocation, job loss and marital breakdown, was positioned as accounting for their greater impact on Lane’s experience of “panic”, rather than menopause or bipolar disorder alone.

Other women described specific life events that were positioned as causing bipolar mood episodes. For example, Olivia described the impact of her marriage breakdown, saying that the stress caused by the relationship ending led to being hospitalised for psychosis because of the impact that it had on her sleep:

And that I found out one weekend that there was actually another woman and I guess I should have known all along and that weekend I just completely lost it… I didn’t sleep that night, I had been taking a Avanza that my GP (General Practitioner) had prescribed. A small amount so that, so I could sleep and I was sleeping really well which had been good but that Friday I didn’t sleep when I found out and then by Saturday I was actually becoming paranoid, delusional…...By the Monday I had – yeah, I was completely <laughs> and then they took me into the emergency department of the private hospital and I was admitted that night.

Oliva also described the process of coming through the other side of this experience: "I had to change my life. I had to actually start doing things that were important to me". She further talked about feeling more at peace after menopause: "I think probably I’ve been the calmest I’ve ever been in my life over the last three years..." and as she described: "I was quite happy to travel in other developing countries in Africa on my own. I had no concern for that. I could calmly make decisions about my life and how it was going". Here, menopause was constructed as a positive life change transition, irrespective of the negative mood that may have occurred during the transition period itself.

Jayce also spoke of the positive “cultural” associations of entering menopause as an Indigenous Australian woman: “I mean, one of the cultural things about menopause is, it is seen as a stage of eldership in the indigenous communities, so I have better relationships with my younger people because I have that status.” Menopause was constructed in this account as being representative of a valued and meaningful phase of life, rather than heralding a set of symptoms to be managed.

For these women, mid-life was positioned as a time of life changes, irrespective of their diagnosis of bipolar disorder or their mood changes. This differentiated menopause from being constructed within a biomedical discourse as a solely a biological period of life, to being constructed as a time of social and personal change.

### Coping with mood change: Treatment choices during menopause

All of women in the sample adopted a biomedical discourse in understanding their bipolar disorder, and were currently taking medication for bipolar disorder, or under management of medical practitioners regarding their symptom management. A biomedical construction of mood changes also provided the framework by which some women chose to engage in treatment for changes in mood during menopause. For the women who positioned menopause as being the cause of symptoms, or being physical in nature, medication such as hormonal-based treatments was sought. For example, Lane reported that she experienced significant sleep disturbances as a result of hot flushes which led her to seek out menopause treatment:

So, the only thing that really worked for me was the HRT. And I tried natural stuff, I’ve tried the progesterone cream that sort of toned them down a bit but still I had the sleeping and the hot flushes still. So HRT has been the only relief and normality of life that I could get with it.

For her, the conceptualisation of menopause as creating a set of specific features or symptoms (hot flushes) that had an impact on her mood led to treatment seeking for menopause to manage the impact of this on bipolar disorder symptoms.

For those women who understood menopause as being secondary to their bipolar disorder, either due to menopause being constructed as ‘natural’ or non-causative, adjustment of psychotropic medication for bipolar symptoms, and psychiatric consultation, was often sought. For example, as noted by Emily:

Since, sort of, this menopause has started, I found my medication wasn’t effective as much, and so they tried increasing it but it didn’t make any difference, and they thought, “Well, let’s try a different anti–depressant,” and so I got switched over to that, and lots of blood tests and things like that. So they obviously – I felt that my symptoms were becoming exaggerated than they were, and they played around with my, sort of, medication to settle it out. And I think I’m now sort of back with the – where I was before menopause.

Here Emily constructs menopause as the cause of changes in her bipolar mood symptoms, with her goal being to return her mood to that which she understood as usual for her. Here the menopause was positioned as having an unusual effect, and within a medical framework this needed to be corrected with medication.

There were some women in the sample who constructed their menopause as a natural life event. As noted by Camilla:

I call it reverse puberty. And instead as far as I am concerned it’s a natural normal progression, and you don’t give kids medication for puberty, so why do you give women medication to get them through menopause. That’s my opinion, anyway.

However psychiatric symptoms were seen as separate from this “natural progression”, and thus, treatment was sought within a medical framework: “I did have to get Valium for a while because I would just be normal and then I would just get this rising panic, even just sitting and watching TV, and I just get this overwhelming panic.” This suggests that some women construct the ‘body’ as being separate from the ‘mind’, with the mind being associated with bipolar disorder, and the body with “natural” menopause. The construction of mood changes as menopausal, or due to life events, also had an influence on women’s evaluation of the outcomes of treatment. As described by Kim:

So, how much is the HRT (Hormone Replacement Therapy) and the menopause ending and things stabilising here and me repairing things with my husband, which bit we can pull out of that as being the HRT, I’d be kind of reluctant to say.

This ambivalence may again reflect that some women in the sample could not separate out causes of mood changes from menopause or bipolar disorder. It may further suggest that some women experienced this phase in a more holistic sense, without clear delineations being obvious between bipolar disorder, stressful life events, menopause and treatment effects. It was also noted by Kim, who was also not on any existing medication for bipolar disorder before menopause, also sought out treatment through alternative therapies, namely traditional Chinese medicine, acupuncture and psychological therapy after trialling HRT:

Because it’s [bipolar disorder] always been relatively low grade and I’m not on medication. I did go on some hormone replacement therapy at one point, which actually did help with sleeping more than anything else which was really helpful. So I tend to use more – I have almost always have had a psychologist and then manage it through that – through the general practice. And for a long time I was taking Chinese herbs, menopause Chinese herbs which – and I also use things like acupuncture to great effect that’s been really helpful along the way.

Here Kim noted that her approach for mood change during this phase menopause was based on her pre-existing management techniques, adding and extending on these in line with her beliefs about self-management and her previous success. This may also indicate that women’s treatment approaches and views of treatment success may change throughout the menopause transition.

In addition to women making decisions about treatment based on their understanding of their mood changes, some also discussed their treatment decisions in relation to other concerns that they had about their bodies during menopause, such as weight gain that can occur as part of the side effects of medications. As Annie stated:

So, I’ve had to go on tablets that made me put on weight quite quickly, put up a whole dress size as well as – and so, when you you’re sort of menopausal, I think, oh, god, I’m gonna – going to put on weight and change shape from that as well….and that’s taken me some time to wrap my head around and get to the point where I felt – well, better to be sane in an extra dress size, than thin and crazy.

Here, although Annie expressed concern about her changing body, she presented the view that her mental health was more important than being “thin”. This may reflect her acceptance of the physical changes if it meant that her mood was more settled, despite being unhappy with the body changes that may have occurred as a result.

## Discussion

This is the first qualitative study that has explored the meaning of menopause for women living with bipolar disorder. Women’s understanding of their mood during menopause was influenced by a number of factors: their experience of bipolar disorder prior to menopause, life events that occurred during mid-life and their construction of mood change as either menopausal or bipolar disorder.

Many women in the sample noted increased mood changes during the menopausal transition that they positioned as resulting from bipolar disorder. This is consistent with previous research that has noted increased mood changes for some women with bipolar disorder during this time [13, 14, 16, 44], and history of depression as a predictor of depression during menopause [29]. However, many women in the sample were reluctant to ascribe increased symptoms to bipolar disorder or menopause, focusing on stressful life events and difficulties in relationships. This is consistent with previous research that has indicated a combination of factors are associated with depressed mood at midlife, including disturbed sleep, sexual problems, poor health, low social support and stressful life events [19, 29, 45, 46].

Many women constructed menopause in terms of changes in ‘the body’, which included vasomotor changes such as hot flushes and sleep disturbances, reflecting dominant cultural constructions of menopausal ‘symptoms’ [24]. However, mood change and other psychiatric symptoms were attributed to the ‘mind’ and seen as separate, yet influenced by vasomotor symptoms. This is consistent with prior research that suggests women make multiple attributions for symptomatology associated with the reproductive life cycle, which can influence their ability to cope [47]. Previous experience of depression has been reported to be a risk factor for depression during menopause [29]. Our findings suggest that previous history of depression may also provide a framework for women to make meaning of mood change during menopause. This has implications for women with bipolar disorder, who may construct menopausal mood changes in a way that is unique to those who have an experience of this illness. This suggests that clinicians and allied health practitioners offering information and support for women during menopause need to be aware of a woman’s experience of mental health issues, as this may influence her negotiation of menopausal symptomatology.

For some women, it was noted that sleep was affected by hot flushes which in turn led to increased negative mood changes during this time, as reported in previous research [29, 30]. Sleep disruptions are also a core feature of bipolar disorder and may be associated with the onset of depression and manic episodes [48], relapse and poorer inter-episode functioning [49]. Given that sleep quality is viewed as critically important in the well-being of those living with bipolar disorder [49], the impact of sleep disruption via hot flushes on women with bipolar disorder needs further attention as this may have a significant impact on how women are able to manage their illness during menopause. This may also be an area that needs to be highlighted in the clinical management of bipolar disorder during menopause, as these symptoms may not be routinely considered during treatment by clinicians. Although there is significant research on the relationship between vasomotor symptoms and mood for women during mid-life [50–53], more work is needed to understand how this may impact on quality of life for women with bipolar disorder. This may inform future psychological interventions in the treatment of bipolar disorder during menopause.

Previous research has reported that those living with bipolar disorder may experience difficulties in a range of areas, whilst others report that bipolar disorder opens up and creates opportunities for them [9]. These findings were supported in this study, where women reported challenges in relation to life events, which for some women led onto new opportunities. This has been noted in prior research on women during mid-life, who report several positive aspects of menopause, including personal growth [54], greater freedom [55], and a sense of new opportunities and experiences opening up [56]. This suggests that life experiences that are predominantly positioned as negative within biomedical discourse, such as bipolar disorder and menopause [57], may be constructed and experienced as positive by women [58]. Research on menopause suggests that these positive constructions are made possible by social and cultural discourse available to women [25], allowing women to adopt a positive subject position in relation to mood change [35]. The availability of positive constructions of bipolar disorder may serve the same purpose for women diagnosed with this illness.

The treatments that women sought during menopause depended on the way in which the women constructed their mood changes, bipolar disorder and medication effects. Those who reported success in managing their illness prior to menopause via medication also sought medication changes during menopause, whereas other women who used alternative means of managing bipolar symptoms sought treatment via these pathways. Previous research has reported that women’s adoption of medical, psychological or self-help treatment menopausal symptoms is influenced by their construction of experience as symptom of illness, or a normal part of midlife transition [59]. There is limited literature that explores treatment pathways during menopause for women living with bipolar disorder, however there are indications that there were no differences between those in early menopause and late menopause in their treatment approach [16]. Clinicians working with women diagnosed with bipolar disorder should be aware of how constructions of their mood changes may impact on the women’s help seeking during this period of life.

There are several points that are to be noted in this study. Firstly, the study consisted of women in Australia and included only one woman who identified as Indigenous. It would be of further interest to explore how women with bipolar disorder from other countries would construct their mood changes during mid-life, and further, how this may then influence their choices regarding treatment. Women with other psychiatric diagnoses such as schizophrenia were not sought in this sample as the focus of the study was bipolar disorder alone. It may be that women living with other psychiatric conditions may construct their mood changes differently than those women living with bipolar disorder. Further research is needed to examine the experience and construction of menopause in women with other psychiatric diagnoses.

One limitation of this study included the small sample size, and it would be worthwhile to further explore this topic in a larger sample of women living with bipolar disorder. Another was the lack of a formal diagnostic interview to confirm bipolar disorder diagnosis and current mood status. Further research could also explore treatment over the life span of women and how women negotiate treatment choices during other key life points such as during the premenstrual phase or pre/post-partum.

## Conclusions

Women with bipolar disorder report a variety of difference experiences during menopause, which may be constructed as menopause, bipolar disorder, or due to life events. Psychosocial interventions have been found to be effective in supporting women with menopausal symptoms [22]. Those working with menopausal women who have bipolar disorder may wish to incorporate elements of these interventions into supportive treatment regimes, to address the complexity of women’s experience of mood change, and need for support, during this time of life.

## Additional file

Additional file 1: Consolidated criteria for reporting qualitative studies. (DOCX 14 kb)

## Abbreviations

GP: General practitioner
HRT: Hormone replacement therapy
STRAW: Stages of reproductive aging workshop
