# Supplementary material for: “Is it menopause or bipolar?”: a qualitative study of the experience of menopause for women with bipolar disorder
Source: BMC Womens Health. 2017 Nov 16;17:110. doi: 10.1186/s12905-017-0467-y (PMC5689207; doi:10.1186/s12905-017-0467-y)
Supplement: Additional file 1: — Consolidated criteria for reporting qualitative studies. (DOCX 14 kb) [file 12905_2017_467_MOESM1_ESM.docx]

**Consolidated criteria for reporting qualitative studies (COREQ): 32-item checklist**

No Item Guide questions/description

Domain 1: Research team and reflexivity

Personal Characteristics

1. Interviewer/facilitator Which author/s conducted the interview or focus group?

Tania Perich

2. Credentials What were the researcher's credentials? E.g. PhD, MD

All authors: PhD

3. Occupation What was their occupation at the time of the study?

First author: Psychologist/Post-doctoral Research Fellow

Second author: Professor

Third author: Post-doctoral Research Fellow

4. Gender Was the researcher male or female?

All authors: Female

5. Experience and training What experience or training did the researcher have?

First author: training in qualitative analysis, no previous qualitative research experience

Second author: significant training and experience in qualitative research spanning over 20 years.

Third author: has worked on several previous qualitative projects and attended training and workshops.

Relationship with participants

6. Relationship established Was a relationship established prior to study commencement?

No – the study was advertised via social media and participants self-selected with the intention of participating in the research.

7. Participant knowledge of the interviewer What did the participants know about the researcher? e.g. personal goals, reasons for doing the research

The participants viewed an online information statement that described the interviewers job role and title.

8. Interviewer characteristics What characteristics were reported about the interviewer/facilitator? e.g. Bias, assumptions, reasons and interests in the research topic

Psychologist working with bipolar disorder, previous research experience.

Domain 2: study design

Theoretical framework

9. Methodological orientation and Theory What methodological orientation was stated to underpin the study? e.g. grounded theory, discourse analysis, ethnography, phenomenology, content analysis

Thematic analysis. Data was viewed from a social constructionist perspective.

Participant selection

10. Sampling How were participants selected? e.g. purposive, convenience, consecutive, snowball

Self-selection sampling

11. Method of approach How were participants approached? e.g. face-to-face, telephone, mail, email

Online survey

12. Sample size How many participants were in the study?

15

13. Non-participation How many people refused to participate or dropped out? Reasons?

Thirty-five women provided contact information for the qualitative interview. Of these, six women were not eligible for the study, because of having reported a diagnosis other than bipolar disorder. The remaining women did not respond to email requests for the interview.

Setting 14. Setting of data collection

Where was the data collected? e.g. home, clinic, workplace

Phone

15. Presence of non-participants Was anyone else present besides the participants and researchers?

No

16. Description of sample What are the important characteristics of the sample? e.g. demographic data, date

Women living with bipolar disorder who have experienced menopause

Data collection

17. Interview guide Were questions, prompts, guides provided by the authors? Was it pilot tested?

Semi-structured interviews were conducted by the first author using the following questions: Can you tell me about your experience of menopause?; what features of menopause do/did you experience?; can you describe how these have/had an impact on you and your life; has menopause changed your bipolar disorder in any way? Follow up questions and prompts were framed using methods described by Magnusson and Marecek (2015), which included questions being phrased as open-ended invitations to offer additional information.

18. Repeat interviews Were repeat interviews carried out? If yes, how many?

No repeat interviews were undertaken

19. Audio/visual recording Did the research use audio or visual recording to collect the data?

Audio recording

20. Field notes Were field notes made during and/or after the interview or focus group?

Field notes were made during the interview

21. Duration What was the duration of the interviews or focus group?

Approximately 30min to 1 hour.

22. Data saturation Was data saturation discussed?

Yes – 3 consecutive interviews

23. Transcripts returned Were transcripts returned to participants for comment and/or correction?

No

Domain 3: analysis and findings

Data analysis

24. Number of data coders How many data coders coded the data?

One

25. Description of the coding tree Did authors provide a description of the coding tree?

Yes

26. Derivation of themes Were themes identified in advance or derived from the data?

Derived from data

27. Software What software, if applicable, was used to manage the data?

Yes – N Vivo

28. Participant checking Did participants provide feedback on the findings?

No

Reporting

29. Quotations presented Were participant quotations presented to illustrate the themes / findings? Was each quotation identified? e.g. participant number

Quotations were used and quotations have been identified by using alternative names to ensure participant privacy.

30. Data and findings consistent Was there consistency between the data presented and the findings?

Yes

31. Clarity of major themes Were major themes clearly presented in the findings?

Yes, these are under headings in the results section

32. Clarity of minor themes Is there a description of diverse cases or discussion of minor themes?

Yes, these are in the results section under the major headings
